# Supplementary material for: A host basal transcription factor is a key component for infection of rice by TALE-carrying bacteria
Source: eLife. 2016 Jul 29;5:e19605. doi: 10.7554/eLife.19605 (PMC4993585; doi:10.7554/eLife.19605)
Supplement: Figure 2—source data 1. — DOI: http://dx.doi.org/10.7554/eLife.19605.007 [file elife-19605-fig2-data1.doc]

0

1

2

3

4

5

6

7

8

9

10

11

12

13

14

15

16

17

18

19

20

21

22

23

23.5

TS

RR

TFB

NLS

AD

-COOH

NH_2_-

**A**

**Figure 2—source data 1.** The defined domains/motifs and sequences of TALE PthXo1 from *Xoo* strain PXO99. (**A**) The conserved structures of PthXo1. TS, amino-terminal translocation signal; RR, central repeat region; TFB, transcription factor binding region; NLS, nuclear localization signal; AD, carboxyl-terminal transcription activation domain. (**B**) The amino acid sequence of PthXo1. The sequence definitions of TS, RR, NLS, and AD are mainly based on a previous study (Yang et al., 2006, Proc. Natl. Acad. Sci. USA 103:10503-10508). The repeat-variable di-residues (RVD), which bind to TALE-binding motif of rice susceptibility gene *Os8N3* promoter based on RVD-DNA binding code specificity, in the RR are shown in blue color. The leucine residues in TFB region are highlighted with yellow color.

**B**

TS

RR

TFB

NLS

AD

Repeat number

Amino acid position
