# Supplementary material for: A host basal transcription factor is a key component for infection of rice by TALE-carrying bacteria
Source: eLife. 2016 Jul 29;5:e19605. doi: 10.7554/eLife.19605 (PMC4993585; doi:10.7554/eLife.19605)
Supplement: Figure 2—source data 2. — DOI: http://dx.doi.org/10.7554/eLife.19605.008 [file elife-19605-fig2-data2.doc]

***Xoc* strain (RH3)**

***Xoo* strain (PXO99)**

TFIIAγ5

TFIIAγ5^V39E^

TFB19

TFB25

TFB12

TFB_Tal9b_/TFB11

TFB22

TFB13

TFB18

TFB_Tal12_/TFB27

TFB17

TFB21

TFB20

TFB16

TFB14

TFB_Tal11a_/TFB15

TFB_Tal3c_/TFB24

TFB26

TFB_Tal5b_/TFB23

TFB28

**Figure 2—source data 2.** Amino acid sequence alignment of the TFB regions of TALEs from *Xanthomonas oryzae* strains, composed of either 134 or 145 amino acids*.* The TFB regions of Tal7b and Tal8b of *Xoo* PXO99 have identical sequences. The leucine residues of PthXo7 are highlighted with red color. None of the TFB regions used for examination of their interaction with mutant TFIIAγ5^V39E^ interact with TFIIAγ1. *Xoo*, *Xanthomonas oryzae* pv. *oryzae*; *Xoc*, *Xanthomonas oryzae* pv. *oryzicola*. H, high affinity interaction; L, low affinity interaction; N, no interaction; nd, not done.

PthXo7/Tal1

Tal9d

AvrXa23/Tal9b

PthXo6/Tal5b

PthXo1/Tal2b

Tal7b (Tal8b)

Tal6a

Tal8a

Tal9a

**TALE**

Tal7a

Tal6b

Tal4

Tal3a

Tal9e

H nd

H nd

L N

H N

H nd

L nd

L nd

H N

H nd

H nd

H nd

H N

H N

H N

H nd

L N

H nd

H N

H N

H N

H N

H L

H N

L N

H H

L N

H H

L N

L N

H N

H N

**SIVAQLSRPDPALAALTNDHLVALACLGGRPALDAVKKGLPHAPELIRRINRRIPERTSH-----------RVPDLAHVVRVLGFFQSHSHPAQAFDDAMTQFEMSRHGLVQLFRRVGVTEFEARYGTLPPASQRWDRILQASGM**

Interact with

**1 10 20 30 40 50 60 70 80 90 100 110 120 130 140 145**

**---------+---------+---------+---------+---------+---------+---------+---------+---------+---------+---------+---------+---------+---------+-----**

**............................................................-----------....Y.................Y...E.........N.....................................**

**............................................N......S..G.....-----------......................H...E.........N.....................................**

**......................................................G.....-----------....Y.................Y...E.........N.....................................**

**............................................................-----------......H.....G...S.....................................C...................**

**............................................................-----------....Y.................Y...E.........N.............F...C...................**

**............................................................RVADLPERTSH......H.....G...S.................................F...C...................**

**.......C....................................................RVADLPERTSN............G...S.....................................C...................**

**............................................................-----------......H.....G...S.....................................C...................**

**...................................................S..G.....-----------....Y.................Y...E.......................F...C...................**

**............................................................-----------H.P...H.....G...S...................N.....................................**

**...................................................S..G.....-----------......H.....G...S...................N.............F...C..................T**

**...................................................S..G.....-----------....Y.................Y...E.........N.....................................**

**............................................................-----------....Y.................Y...E.........N.....................................**

**............................................................-----------....................................N..L.......A..........................**

**.............T..............................................-----------......................................................C..........D........**

**............................................................-----------....................................Q..L.......A..........................**

**............................................................-----------....................................Q..L..................................**

**............................................................-----------................S..Q..Q...D..........................S....................**

**............................................................-----------....................................N..L........................R.........**

**............................................................-----------....................................N..L..................................**

**.............................................F........A.....-----------......................Q...D.......................F...Y..........D........**

**...................................................R........-----------................S..Q..Q...D..........................S....................**

**........R..........................................R........-----------................S..Q..Q...D..........................S....................**

**...................................................R........-----------....................................Q.................C..........D........**

**.............................................F........A.....-----------......................................................C..........D........**

**............................................................-----------................S..Q..Q...D..........................S....................**

**............................................................-----------......H...................................................................**

**.............................................F.....R........-----------......H.............................N..L..............C..........D........**

**............................................................-----------......................................................C..........D........**

**.............................................F.....R..P.....-----------......H...........................................F...Y..........D........**

**.............................................F.....R..A.....-----------..........................................................................**

H nd
