# Supplementary material for: A host basal transcription factor is a key component for infection of rice by TALE-carrying bacteria
Source: eLife. 2016 Jul 29;5:e19605. doi: 10.7554/eLife.19605 (PMC4993585; doi:10.7554/eLife.19605)
Supplement: Figure 3—source data 1. — DOI: http://dx.doi.org/10.7554/eLife.19605.011 [file elife-19605-fig3-data1.doc]

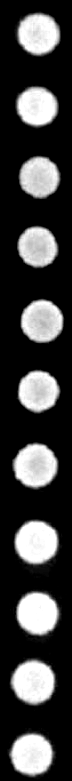

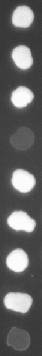


**Lesion length (cm)**


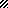

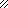

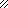

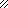

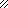

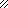


TFB-m1

TFB-m3

TFB-m1

TFB-m3


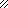

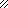

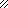

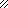

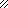


TFB^PthXo1^

SIVAQ**L**SRPDPA**L**AA**L**TNDH**L**VA**L**AC**L**GGRPAMDAVKKG**L**PHAPE**L**IRRV

NRRIGERTSHRVADYAQVVRV**L**EFFQCHSHPAYAFDEAMTQFGMSRNG**L**V

Q**L**FRRVGVTE**L**EARGGT**L**PPASQRWDRI**L**QASGM

TFB-m1

SIVAQ**A**SRPDPA**A**AA**A**TNDH**A**VA**A**AC**A**GGRPAMDAVKKG**A**PHAPE**A**IRRV

NRRIGERTSHRVADYAQVVRVLEFFQCHSHPAYAFDEAMTQFGMSRNGLV

QLFRRVGVTELEARGGTLPPASQRWDRILQASGM

TFB-m2

SIVAQLSRPDPALAALTNDHLVALACLGGRPAMDAVKKGLPHAPELIRRV

NRRIGERTSHRVADYAQVVRV**A**EFFQCHSHPAYAFDEAMTQFGMSRNG**A**V

Q**A**FRRVGVTE**A**EARGGT**A**PPASQRWDRI**A**QASGM

TFB-m3

SIVAQ**A**SRPDPA**A**AA**A**TNDH**A**VA**A**AC**A**GGRPAMDAVKKG**A**PHAPE**A**IRRV

NRRIGERTSHRVADYAQVVRV**A**EFFQCHSHPAYAFDEAMTQFGMSRNG**A**V

Q**A**FRRVGVTE**A**EARGGT**A**PPASQRWDRI**A**QASGM

**Figure 3—source data 1.** Effects of leucine residues of PthXo1 TFB region on TALE-mediated infection. (**A**) Mutation of the leucine (L) residues (blue colored) of PthXol TFB region (TFB^PthXo1^) with alanine (A, red colored). (**B**) Mutation of the leucine residues did not influence the interaction between mutated TFB regions and TFIIAγ5 in yeast cells. The interactions were assessed by growth of yeast cells on synthetic defined premixes (SD) medium lacking (-) leucine (L), tryptophan (W), histidine (H), and adenine (A). (**C**) Mutation of leucine residues of PthXo1 TFB region did not change the virulence of *Xoo* strains in near-isogenic lines IR24 and IRBB5 compared to strain T7174(PthXo1-TFB^PthXo1^) or KACC10331(PthXo1-TFB^PthXo1^). Each bar represents mean (total 35 to 40 leaves from 5 plants) ± standard deviation. b, significant difference between wild-type T7174 or KACC10331 and recombinant strains in each rice line at *P* < 0.01.

**A**

IR24 (*TFIIAγ5*)

IRBB5 (*TFIIAγ5^V39E^*)

b

**C**

T7174(PthXo1-TFB)

0

5

10

15

20

25

30

b

T7174

TFB^PthXo1^

TFB-m2

b

KACC10331 (PthXo1-TFB)

0

5

10

15

b

TFB^PthXo1^

TFB-m2

KACC10331

b

b

b

b

b

b

b

b

b

b

b

b

***Xoo* strain**

***Xoo* strain**

**B**

SD/-LW

p53 (control) RecT

lamin C (control) RecT

TFB^PthXo1^(wild type) TFIIAγ5

DNA-binding domain vector

Activation domain vector

SD/-LWHA

TFB-m1 TFIIAγ5

TFB-m2 TFIIAγ5

TFB-m3 TFIIAγ5
