# Supplementary material for: A host basal transcription factor is a key component for infection of rice by TALE-carrying bacteria
Source: eLife. 2016 Jul 29;5:e19605. doi: 10.7554/eLife.19605 (PMC4993585; doi:10.7554/eLife.19605)
Supplement: Figure 4—source data 1. — DOI: http://dx.doi.org/10.7554/eLife.19605.014 [file elife-19605-fig4-data1.doc]

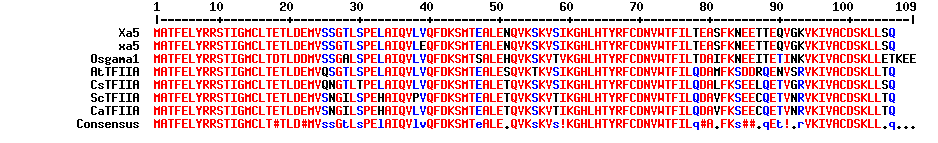


**Figure 4—source data 1.** Amino acid sequence alignment of basal transcription factor IIA gamma subunit (TFIIAγ) from different species. The 39^th^ and 47^th^ residues of TFIIAγ5 are highlighted with red color. The dot indicates an identical amino acid residue. The dash (-) indicates lack of an amino acid residue. TFIIAγ5/XA5 (accession number in protein database or GenBank of National Center for Biotechnology Information [http://www.ncbi.nlm.nih.gov]: AAO33769), TFIIAγ5^V39E^/xa5 (AAV53715), and TFIIAγ1 (NP_001045526) from *Oryza sativa*; AtTFIIAγ (Q39236) from *Arabidopsis thaliana*; CsTFIIAγ (XP_006433782) from *Citrus sinensis*; SlTFIIAγ (XP_004252721) from *Solanum lycopersicum*; CaTFIIAγ (KU163013) from *Capsicum annuum*; HsTFIIAγ (XP_510452) from *Homo sapiens*; TOA2 (AAB58717) from *Rattus norvegicus*; VvTFIIAγ (XP_002285630) from *Vitis vinifera*; ZmTFIIAγ (ACG32599) from *Zea mays*; SbTFIIAγ (XP_002440415) from *Sorghum bicolor*; MtTFIIAγ (XP_003620976) from *Medicago truncatula*; GmTFIIAγ (XP_003519205) from *Glycine max*; DrTFIIAγ (NP_001018441) from *Danio rerio*; BdTFIIAγ (XP_003569111) from *Brachypodium distachyon*; DmTFIIAγ (NP_524467) from *Drosophila melanogaster*.

**MATFELYRRSTIGMCLTETLDEMVSSGTLSPELAIQVLVQFDKSMTEALENQVKSKVSIK-GHLHTYRFCDNVWTFILTEASFKNEETTEQVGKVKIVACDSKLLSQ**

**.................D...D.....A..................S...H......TV.-..................D.I........TIN............ETKEE**

**........................QN........................S......T..-.................QD.L.....SQ.N.R............T.**

**.........................N.I....H.................T......T..-.................QD.V..S..CQ.T.NR...........T.**

**.........................N..X.................D..........TV.-..................D.......A.................G.**

**........................QN...T....................T.........-.................QD.L..S..LQ.T..R.............**

**..-YQ...NT...NS.Q.S...LIQTQQIT.Q..L...L....AINT..A.R.RNR.NFR-GS.N...........V.NDVE.REVTDLVK.D........G.NTGSNAAE**

**.........................................................TV.-..................D.Q.......................T.**

**........................QN........................T.........-.................QD.L....DNQ.N..R.............**

**..............................................D..........N..-..................D.........................G.**

**.........................N.I....H.................T......T..-...........V.....QD.V..S..CQ.T.NR...........T.**

**.S-YQ...NT...NT.Q.S...LIQY.QIT.G..FK..L.....INN..NQR..AR.TF.A.K.N..........LM.NDVE.REVHEIVK.D........GKSGEF**

**..-YQ...NT...NS.Q.S...LIQ.QQIT.Q..L...L....AINA..AQR.RNR.NFR-.S.N...........V.NDVE.REVTELIK.D........G.NTGSNTTE**

**..-YQ...NT.L.NS.Q.S...LIQ.QQIT.Q..L...L....AINS..AQR.RNR.NFR-.S.N...........V.NDVE.REVTELIK.D........G.NTGSNTTE**

**......................................E.....................-..............................................**

**.........................N.I....H.................T......T..-...........V.....QD.V..S..CQ.T.NR...........T.**

**........................QN........................T.........-.................QD.L....DSQ.N..R...........T.**

**........................Q.........................S...T.....-.................QD.M..SDDRQ.N.SR...........T.**

106

106

106

109

106

106

109

106

109

106

106

109

106

106

106

106

106

106

TFIIAγ5^V39E^

VvTFIIAγ

SlTFIIAγ

TOA2

ZmTFIIAγ

CaTFIIAγ

HsTFIIAγ

SlTFIIAγ

TFIIAγ1

SbTFIIAγ

MtTFIIAγ

DrTFIIAγ

BdTFIIAγ

DmTFIIAγ

CsTFIIAγ

TFIIAγ5

GmTFIIAγ

AtTFIIAγ
