# Supplementary material for: A host basal transcription factor is a key component for infection of rice by TALE-carrying bacteria
Source: eLife. 2016 Jul 29;5:e19605. doi: 10.7554/eLife.19605 (PMC4993585; doi:10.7554/eLife.19605)
Supplement: Figure 4—source data 2. — DOI: http://dx.doi.org/10.7554/eLife.19605.015 [file elife-19605-fig4-data2.doc]

42819470

42820786

**Figure 4—source data 2.** Single nucleotide polymorphisms in the *TFIIAγ1* coding region of 1419 rice accessions from

RiceVarMap (http://ricevarmap.ncpgr.cn). These rice accessions encode identical TFIIAγ1.

42819513 T>C

Synonymous

42820700 A>C

Synonymous

Rice classification

Population Size

Frequency of T

(major allele)

Frequency of C

(minor allele)

Frequency of T

(major allele)

Frequency of C

(minor allele)

All

1419

84.43%

15.57%

89.08%

10.92%

All Indica

799

79.97%

20.03%

81.10%

18.90%

Indica I

375

68.80%

31.20%

69.87%

30.13%

Indica II

213

95.31%

4.69%

96.71%

3.29%

Indica

intermediate

211

84.36%

15.64%

85.31%

14.69%

All Japonica

497

97.18%

2.82%

100.00%

0.00%

Temperate

Japonica

335

100.00%

0.00%

100.00%

0.00%

Tropical

Japonica

94

85.11%

14.89%

100.00%

0.00%

Japonica

intermediate

68

100.00%

0.00%

100.00%

0.00%

Aus

67

41.79%

58.21%

100.00%

0.00%

Group VI/Aromatic

14

100.00%

0.00%

100.00%

0.00%

Intermediate

42

80.95%

19.05%

90.48%

9.52%

ATG

TAA
