# Supplementary material for: A host basal transcription factor is a key component for infection of rice by TALE-carrying bacteria
Source: eLife. 2016 Jul 29;5:e19605. doi: 10.7554/eLife.19605 (PMC4993585; doi:10.7554/eLife.19605)
Supplement: Figure 4—source data 3. — DOI: http://dx.doi.org/10.7554/eLife.19605.016 [file elife-19605-fig4-data3.doc]

437482 C>T

Synonymous

437499 T>A

Non

-

Synonymous

437500 C>G

Non

-

Synonymous

Rice classification

Population

size

Frequency of C

(major allele)

Frequency of T

(minor allele)

Frequency of T

(major allele)

Frequency of A

(minor allele)

Frequency of C

(major allele)

Frequency of G

(minor allele)

All

1419

97.53%

2.47%

97.67%

2.33%

97.67%

2.33%

All Indica

799

96.75%

3.25%

99.50%

0.50%

99.50%

0.50%

Indica I

375

100.00%

0.00%

100.00%

0.00%

100.00%

0.00%

Indica II

213

89.67%

10.33%

98.12%

1.88%

98.12%

1.88%

Indica

intermediate

211

98.10%

1.90%

100.00%

0.00%

100.00%

0.00%

All Japonica

497

100.00%

0.00%

100.00%

0.00%

100.00%

0.00%

Temperate

Japonica

335

100.00%

0.00%

100.00%

0.00%

100.00%

0.00%

Tropical

Japonica

94

100.00%

0.00%

100.00%

0.00%

100.00%

0.00%

Japonica

intermediate

68

100.00%

0.00%

100.00%

0.00%

100.00%

0.00%

Aus

67

91.04%

8.96%

56.72%

43.28%

56.72%

43.28%

Group VI/ Aromatic

14

92.86%

7.14%

100.00%

0.00%

100.00%

0.00%

Intermediate

42

95.24%

4.76%

100.00%

0.00%

100.00%

0.00%

ATG

TAA

437384

442893

IR24 (TFIIAγ5) 437498-437500:GTC: encoding valine (V) at 39^th^ residue

IRBB5 (TFIIAγ5^V39E^) 437498-437500:GAG: encoding glutamine (E) at 39^th^ residue

**Figure 4—source data 3.** Single nucleotide polymorphisms in the *TFIIAγ5* coding region of 1419 rice accessions from RiceVarMap ([http://ricevarmap.ncpgr.cn](http://ricevarmap.ncpgr.cn/)). Approximately 2.3% of these rice accessions encode mutated TFIIAγ5 (TFIIAγ5^V39E^) as rice line IRBB5.
