# Supplementary material for: A host basal transcription factor is a key component for infection of rice by TALE-carrying bacteria
Source: eLife. 2016 Jul 29;5:e19605. doi: 10.7554/eLife.19605 (PMC4993585; doi:10.7554/eLife.19605)
Supplement: Supplementary file 1. — DOI: http://dx.doi.org/10.7554/eLife.19605.025 [file elife-19605-supp1.doc]

**Supplementary file 1.** Measurements of agronomic traits of rice lines IR24 and IRBB5 under natural field conditions

| Phenotype | IR24 (*TFIIAγ5*) | IRBB5 (*TFIIAγ5^V39E^*)^1^ |
| --- | --- | --- |
| Heading date (days) | 98.00 ± 2.00 | 97.50 ± 3.50 |
| Plant height (cm) | 95.22 ± 3.51 | 86.42 ± 2.38^b^ |
| Flag leaf length (cm) | 41.30 ± 1.23 | 41.42 ± 1.32 |
| Number of panicles per plant | 16.00 ± 1.60 | 16.40 ± 1.10 |
| Panicle length (cm) | 24.32 ± 0.83 | 24.52 ± 0.47 |
| Grains per panicle | 198.50 ± 8.00 | 199.50 ± 7.00 |
| 1000-grain weight (g) | 24.80 ± 1.78 | 24.73 ± 1.27 |
| Yield per plant (g) | 32.15 ± 4.30 | 32.00 ± 2.30 |
| Seed set (%) | 90.40 ± 4.30 | 90.10 ± 1.60 |
| Grown grain length (mm) | 6.34 ± 0.13 | 6.35 ± 0.16 |
| Grown grain width (mm) | 2.13 ± 0.09 | 2.12 ± 0.17 |
| Grown grain thickness (mm) | 1.85 ± 0.22 | 1.83 ± 0.17 |

^1^The “b" indicates a significant difference between IR24 and IRBB5 at *P* < 0.01.
