# Supplementary material for: A host basal transcription factor is a key component for infection of rice by TALE-carrying bacteria
Source: eLife. 2016 Jul 29;5:e19605. doi: 10.7554/eLife.19605 (PMC4993585; doi:10.7554/eLife.19605)
Supplement: Supplementary file 2. — DOI: http://dx.doi.org/10.7554/eLife.19605.026 [file elife-19605-supp2.doc]

**Supplementary file 2.** PCR primers used for construction of vectors for protein–protein interactions and transformation, and detection of positive transgenic plants

^a^The underlined nucleotides are the digestion site of *Spe*I.

| Gene (accession number) | Forward primer (5’-3’) | Reverse primer (5’-3’) | Product size (nt) | Use |
| --- | --- | --- | --- | --- |
| *TFIIAγ5/Xa5* (AK065182) | AGACTAGT^a^GGTACC^b^ATTGATAACTGCGAGGTCAG | AAGAGCTC^c^GGATCC^d^TACATACAAAGAGTCACACAG | 312 | Amplifying cDNA fragment for constructing RNAi construct |
|  | CCGGAATTC^e^ATGGCCACCTTCGAGCTC | CGCGGATCC^d^TTATTGGCTGAGTAGTTTG | 321 | Amplifying cDNA fragment for constructing yeast two hybrid construct |
|  | CGGGGTACC^b^ATGGCCACCTTCGAGCTCTACCGGAGG | CGCGGATCC^d^TTGGCTGAGTAGTTTGGAATCACAGG | 318 | Amplifying cDNA fragment for constructing expressing construct |
| *TFIIAγ1* (CB097192) | AGACTAGT^a^GGTACC^b^AAAGAAGAGTAATTTGCGG | AAGAGCTC^c^GGATCC^d^GAGCACACTCATGTCACAC | 269 | Amplifying cDNA fragment for constructing RNAi construct |
|  | CCGGAATTC^e^ATGGCCACCTTCGAGCTC | CGCGGATCC^d^TTACTCTTCTTTAGTCTC | 330 | Amplifying cDNA fragment for constructing yeast two hybrid construct |
|  | CGGGGTACC^b^ATGGCCACCTTCGAGCTGTACCGGAGG | CGCGGATCC^d^CTCTTCTTTAGTCTCCAGCAATTTGG | 327 | Amplifying cDNA fragment for constructing expressing construct |
|  | CGGGGTACC^b^ATGGCCACCTTCGAGCTGTACCGGAGG | CACCTGATGCTCCAAAGCCTCAGTCATGG | 159 | Amplifying cDNA fragment for constructing site-directed mutation expressing construct |
|  | GCTTTGGAGCATCAGGTGAAGAGCAAGG | CGCGGATCC^d^CTCTTCTTTAGTCTCCAGCAATTTGG | 189 | Amplifying cDNA fragment for constructing site-directed mutation expressing construct |
| *TFIIAαβ* (AK065373) | CCGGAATTC^e^ATGGCCAGCAGCAACGTCTCCACG | CGCGGATCC^d^TCAAAAATCAAATTCGCCGGTAGC | 1185 | Amplifying cDNA fragment for constructing yeast two hybrid construct |
| *pthXo1* (CP000967) | CCCAAGCTT^g^GGTGTGTTTGGTGAATTTTGCAG | GCTCTAGA^h^TTGTCTCAAGGCGCAGAAATGATCT | 5487 | Amplifying DNA fragment |
|  | CCGGAATTC^e^GTGCAATCGGGTCTGCG | GCGTCGAC^i^GGTTCAGGGGGGCACCCGT | 546 | Amplifying DNA fragment encoding TS of PthXo1 |
|  | GCGTCGAC^i^AGAGCATTGTTGCCCAG | AACTGCAG^j^GTCAGATCGTCCCTCCGAC | 861 | Amplifying DNA fragment encoding from TFB to AD of PthXo1 |
|  | GCGTCGAC^i^AGAGCATTGTTGCCCAG | AACTGCAG^j^GCCACATCACGGTGCTGG | 741 | Amplifying DNA fragment encoding from TFB to NLS of PthXo1 |
| *TFB* | CCGGAATTC^e^AGCATTGTTGCCCAGTTATCTCG | CGCGGATCC^d^CATCCCTGATGCCTGGAGGATAC | 402 | Amplifying DNA fragment from different TALEs for constructing yeast two hybrid constructs |
|  | CGGGGTACC^b^ATGAGCATTGTTGCCCAGTTATC | TCCCCCGGG^k^CATCCCTGATGCCTGGAGGATAGC | 405 | Amplifying DNA fragment from different TALEs for constructing expressing construct |
| *9myc* (AY788908) | TCCCCCGGG^k^GGTGAACAAAAGTTGATTTCTGAAG | GGAAGATCT^l^TCATCCGTTCAAGTCTTCTTCTGAG | 351 | Amplifying DNA fragment for constructing expressing construct |

^b^The underlined nucleotides are the digestion site of *Kpn*I.

^c^The underlined nucleotides are the digestion site of *Sac*I.

^d^The underlined nucleotides are the digestion site of *Bam*HI.

^e^The underlined nucleotides are the digestion site of *Eco*RI.

^f^The underlined nucleotides are the digestion site of *Xho*I.

^g^The underlined nucleotides are the digestion site of *Hin*dIII.

^h^The underlined nucleotides are the digestion site of *Xba*I.

^i^The underlined nucleotides are the digestion site of *Sal*I.

^j^The underlined nucleotides are the digestion site of *Pst*I.

^k^The underlined nucleotides are the digestion site of *Sma*I.

^l^The underlined nucleotides are the digestion site of *Bgl*II.
