# Supplementary material for: A host basal transcription factor is a key component for infection of rice by TALE-carrying bacteria
Source: eLife. 2016 Jul 29;5:e19605. doi: 10.7554/eLife.19605 (PMC4993585; doi:10.7554/eLife.19605)
Supplement: Supplementary file 3. — DOI: http://dx.doi.org/10.7554/eLife.19605.027 [file elife-19605-supp3.doc]

**Supplementary file 3. PCR primers used for quantitative RT-PCR assays**

| Gene (GenBank accession no.) | Forward primer (5’-3’) | Reverse primer (5’-3’) | Product size (nt) |
| --- | --- | --- | --- |
| Os*8N3*  (DQ421395) | TGGTTCTGCTACGGCCTCTT | GGTACCAGAAGTAGAGCCCCATCT | 103 |
| *TFIIAγ5/Xa5* (AK065182) | TGGCCACCTTCGAGCTCTA | CTCGTCGAGCGTCTCAGTGA | 101 |
| Os*11N3* (AK101913) | ATCTACTACGCGCTGCTCAAGTC | TAGACGAGGTAGACGGCGATGT | 100 |
| *TFIIAγ1* (CB097192) | CGAGCTCGCCATCCAAGT | TGTGCAGATGGCCCTTGAC | 101 |
| Os*TFX1* (AK108319) | CCCACTACCACAGCAACATGA | CACAGGTAGCTGCTGGGAAGT | 101 |
| *OsSULTR3;6* (AK121195) | TGGCGATGGTCAAGAACGA | TGATACCAAACGCGATCATCTC | 60 |
| *actin*  (X15865) | TGTATGCCAGTGGTCGTACCA | CCAGCAAGGTCGAGACGAA | 121 |
